# Lack of p53 function promotes radiation-induced mitotic catastrophe in mouse embryonic fibroblast cells

**DOI:** 10.1186/1475-2867-6-11

**Published:** 2006-04-26

**Authors:** Fiorenza Ianzini, Alessandro Bertoldo, Elizabeth A Kosmacek, Stacia L Phillips, Michael A Mackey

**Affiliations:** 1 Departments of Pathology, Radiation Oncology, and Biomedical Engineering, University of Iowa, Iowa City, IA 52242, USA; 2 Department of Biomedical Engineering, University of Iowa, Iowa City, IA 52242, USA; 3 Departments of Biomedical Engineering and Pathology, University of Iowa, Iowa City, IA 52242, USA

## Abstract

**Background:**

We have demonstrated that in some human cancer cells both chronic mild heat and ionizing radiation exposures induce a transient block in S and G2 phases of the cell cycle. During this delay, cyclin B1 protein accumulates to supranormal levels, cyclin B1-dependent kinase is activated, and abrogation of the G2/M checkpoint control occurs resulting in mitotic catastrophe (MC).

**Results:**

Using syngenic mouse embryonic fibroblasts (MEF) with wild-type or mutant p53, we now show that, while both cell lines exhibit delays in S/G2 phase post-irradiation, the mutant p53 cells show elevated levels of cyclin B1 followed by MC, while the wild-type p53 cells present both a lower accumulation of cyclin B1 and a lower frequency of MC.

**Conclusion:**

These results are in line with studies reporting the role of p53 as a post-transcriptional regulator of cyclin B1 protein and confirm that dysregulation of cyclin B1 promote radiation-induced MC. These findings might be exploited to design strategies to augment the yield of MC in tumor cells that are resistant to radiation-induced apoptosis.

## Introduction

Mitotic catastrophe (MC) has been observed following alterations in specific cellular proteins, or by treatment of cells with chemicals, heat, and/or ionizing radiation [1-13]. MC is characterized by an aberrant nuclear morphology observed following premature entry into mitosis [10] and often results in the generation of aneuploid and polyploid cell progeny. The initiating event in this process involves the premature entry of cells into mitosis; cells undergo a spontaneous chromosome condensation that produces chromosome morphologies very similar to those observed when metaphase cells are fused with cells located late in the cell cycle [11]. Thus we consider these abnormal mitotic figures as indicative of cells undergoing spontaneous premature chromosome condensation (SPCC). These cells entering into mitosis prematurely often either fail to achieve cytokinesis or divide and fuse shortly thereafter, and later exhibit the features of MC. These cells almost always die [9]; however, some studies have suggested that a small fraction of cells might survive long enough to establish a growing population of cells [14-16], and one study demonstrated a high frequency of surviving clones containing an elevated incidence of MC [17]. These results may indicate that a small fraction of cells can survive MC.

Stress-induced SPCC and subsequent MC is observed under conditions where cyclin B1/cdc2 kinase is activated while cells are delayed in S or G2 phases [20,11], indicating that stress-induced MC is the result of abrogation of cell cycle regulatory pathways, in particular G2 checkpoint pathways [1,21,22]. There are many proteins that play a role in the regulation of checkpoint functions in G2, both inhibitory and stimulatory. Our previous work has shown that significant elevations of the intracellular content of cyclin B1 are associated with SPCC and subsequent MC under a variety of conditions [11,13,20]. Thus, there is evidence in support of the hypothesis that abrogation of the G2/M checkpoint, due to overaccumulation of cyclin B1 protein and premature activation of cyclin B1/cdc2 kinase, plays a critical role in the induction of SPCC and subsequent MC.

Cyclin B1 biosynthesis contributes to the regulation of mitotic entry, as cyclin B1 levels are cell cycle regulated, with the gene being expressed only in S and G2 phases in human and rodent cells [25]. At the later stages of mitosis, proteosome-mediated degradation of cyclin B1 begins, and new cyclin B1 synthesis is required for entry into the next mitosis [26]. Thus, the cyclic rise and fall of cyclin B1 levels provides for one level of regulation of this pro-mitotic protein. Cells arrested late in the cell cycle are located at that point in the cycle when cyclin B1 gene expression is at its peak value. Under these conditions it has been shown that the p53 tumor suppressor gene product is a negative regulator of cyclin B1 transcription, perhaps providing for negative feedback regulation of cyclin B1 levels under abnormal conditions [27]. If the induction of MC in cells post-irradiation is due to cyclin B1 overaccumulation, a role for p53 in this response might be expected. In this study we present data which describe such a role for p53 in the induction of MC mediated by overaccumulation of cyclin B1 occurring during delay of cells late in the cell cycle.

## Materials and methods

### Cell culture

Mouse embryonic fibroblast (MEF) cells were grown in monolayer in Dulbecco's modified eagle medium (DMEM) (GIBCO) containing 10% heat-inactivated fetal bovine serum (Hyclone), non-essential aminoacids (GIBCO) and antibiotics (100 U/ml penicillin and 100 μg/ml streptomycin) (GIBCO). Under these growth conditions, cells grew with a doubling time of about 14 hours. Cells were routinely tested for mycoplasma and found to be uninfected. For all experiments, cells were plated at a density of 1.5 × 10^6 ^*per *75 cm^2 ^flask 24 hours prior to the start of the experiment.

### Irradiation

Gamma-irradiation was delivered at room temperature using a 9,000 Ci ^137^Cs source at the dose rate of 0.92 Gy min^-1^.

### Cytology

Microscopic determination of nuclear fragmentation was performed on air-dried slide preparations. Hypotonically swollen cells (75 mM KCl, 8 min) were fixed in 3:1 absolute methanol to glacial acetic acid for 5 min, and air-dried on clean, moistened microscopic slides. Dried slides were then stored at room temperature until ready for staining with 1 mg/ml Hoechst 33342. The percentage of cells exhibiting nuclear fragmentation was determined using an Olympus AX-70 microscope equipped with a mercury lamp and ultraviolet filters. Only intact cells containing three or more fragmented nuclei were scored for these experiments.

### Light microscopy

The same microscopic slides prepared for the cytology end point (described above) were used to depict cell morphology changes post-irradiation. Photos were taken using an epifluorescence Olympus BX51 microscope at a magnification of 10×.

### Bivariate BrdUrd-PI (bromodeoxiuridine-propidium iodide) flow cytometry

Analysis of cell cycle distribution during the post-irradiation interval was determined using 10 μM BrdUrd pulse-labeling techniques, followed by bivariate analysis using anti-BrdUrd-PI staining (Becton Dickinson Immunocytometry Systems), as previously described in Ianzini and Mackey 1997 [11] to monitor cells in G1, S, and G2 phases. Flow cytometric analysis was performed using a FacSTAR (Becton Dickinson), with excitation of fluorochromes by an argon laser emitting at 488 nm with 300 mW power; red fluorescence (PI) was detected using a 640 nm low-pass filter, and green fluorescence (FITC) using a 525 nm band-pass filter. Data analysis was performed using WinMDI software. For all the collected data, cell cycle phase distributions were determined using box analysis as described in detail in Ianzini and Mackey 1997 [11].

### Bivariate cyclin B1-PI flow cytometry

Estimates of the relative intracellular levels of cyclin B1 were made using anti-cyclin B1-PI analysis as described in Mackey and Ianzini 2000 [13]. Briefly, cells fixed in 95% ethanol were incubated (1 hour, room temperature) with an anti-cyclin B1 monoclonal antibody (Upstate Biotechnology), rinsed and incubated as before with FITC-conjugated goat-anti-mouse IgG (Sigma), treated with RNAse (1 mg/ml, 30 min, room temperature) after which 0.5 ml of 70 μg/ml of PI was added. The details for flow cytometric data acquisition were the same as for the anti-BrdUrd-PI analysis above. In all samples analyzed, cyclin fluorescence was detected only in cells with early S DNA content. The WinMDI software was used to determine the mean cyclin fluorescence values per cyclin-labeled cells; these measurements are proportional to intracellular cyclin levels.

### Western blotting

Western Blotting was performed to determine cyclin B1 protein expression in the mutant p53 MEF 10(1) cells and in the wild-type p53 MEF 12(1) cells. Thirty μg total proteins from whole cell extracts were boiled for 10 min in Laemmli sample buffer and separated using 10–12% 1D SDS-PAGE. The separated proteins were transferred to Immobilon-P membranes using a semi-dry blotting apparatus and probed with an anti-cyclin B1 monoclonal antibody (anti-mouse) (Upstate), at a dilution of 1:500. Beta-actin was detected using goat anti-actin polyclonal antibody (Santa Cruz Biotechnology Inc.). Peroxidase-conjugated AffiniPure goat anti-mouse IgG Fcy fragment specific (Jackson ImmunoResearch) and donkey anti-goat IgG HRP (Santa Cruz Biotechnology) were used as secondary antibodies at a dilution of 1:6000 and 1:2000, respectively. Detection was performed using Western Lightning Chemiluminescence Reagent (Perkin Elmer).

## Results

To explore a role for p53 in the induction of mitotic catastrophe (MC) by radiation, we studied the response to γ-irradiation of both wild-type and non-functional p53 mouse embryonic fibroblast (MEF) cells. These two related cell lines are the parental MEF 12(1) (wild-type p53) and MEF 10(1) (mutant p53). These cell lines were originally developed in Dr. A. J. Levine's lab (Princeton University) and were kindly provided to us by Dr. P. Goswami (University of Iowa). p53 DNA binding assays have demonstrated normal p53 DNA binding activity for the wild-type 12(1) cell line, with no p53 binding for the p53 mutant MEF 10(1) cells (Dr. Goswami, personal communication).

The data presented here are from a representative experiment; all results were verified in two additional experiments. For each experiment, control and treated samples were collected at the same time with matching time points for each end point.

**Figure 1:**
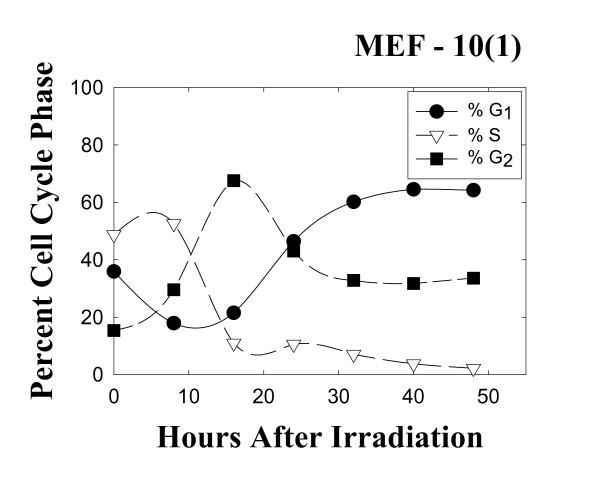
Changes in the cell cycle phase distribution of the mutant p53 cell line MEF 10(1) following 10 Gy γ-irradiation. Cells were pulse-labeled for 30 min with 10 μM BrdUrd in complete medium at 37°C at the indicated times post-irradiation. Bivariate BrdUrd-PI flow cytometric analysis was performed as described in Materials and Methods. 20000 cells were analyzed at each time point. Closed circles, percentage of cells in G1 phase of the cell cycle; open triangles, percentage of cells in S phase of the cell cycle; closed squares, percentage of cells in G2 phase of the cell cycle.

Figure 1 shows changes in cell cycle phase distribution following 10 Gy irradiation of the mutant p53 MEF 10(1) cell line. Following irradiation, there is a transient decrease in G1 fraction, while the S phase fraction stays around 50% for about 8 hours. Following these effects on G1 and S fractions, a transient increase in the G2 fraction is observed, while the S phase fraction remains depressed for the duration of the experiment. These data are in accord with other studies [11] and they show the expected delay in G2 phase, which peaks at about 18 hours post-irradiation. Sham-irradiated controls showed no effect of cell manipulation on cell cycle phase distribution (data not shown).

**Figure 2:**
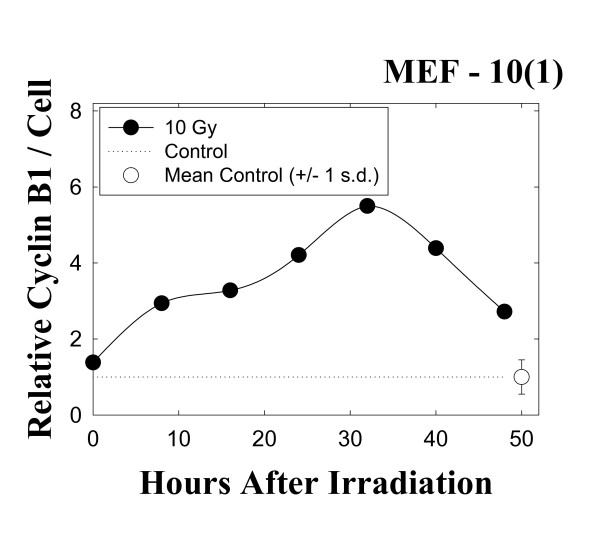
Changes in intracellular cyclin B1 levels in the mutant p53 cell line MEF 10(1) following 10 Gy γ-irradiation. Cyclin B1 levels were determined using a bivariate flow cytometric assay, as described in Materials and Methods, and are expressed relative to sham-irradiated controls. The open circle at the end of the dotted line shows the mean and standard deviation of cyclin levels in the matched controls over the course of this experiment. Cyclin B1 was detected only in early S phase.

Our previous work [11,13,20] has shown that cells delayed late in the cell cycle contain elevated levels of cyclin B1. Thus, we performed flow-cytometric determinations of cyclin B1 levels using immunofluorescence staining with a cyclin B1 antibody and counterstaining with propidium iodide (Figure 2). This bivariate analysis [13] allows us to determine the relative cyclin B1 levels as a function of DNA content, as described in detail in Mackey and Ianzini [13]. As this assay measures cyclin B1 levels *per *cell, points above the dotted line in Figure 2 represent cells with abnormal accumulations of cyclin B1. As can be seen in Figure 2, cyclin B1 begins to rise immediately following irradiation, and continues to increase as cells are later delayed in G2. Cyclin B1 levels are already 3 times the control value at 8 hours post-irradiation. This elevation in cyclin B1 content begins to decrease at 34 hours post-irradiation, but still remains above control levels for the duration of the experiment.

**Figure 3:**
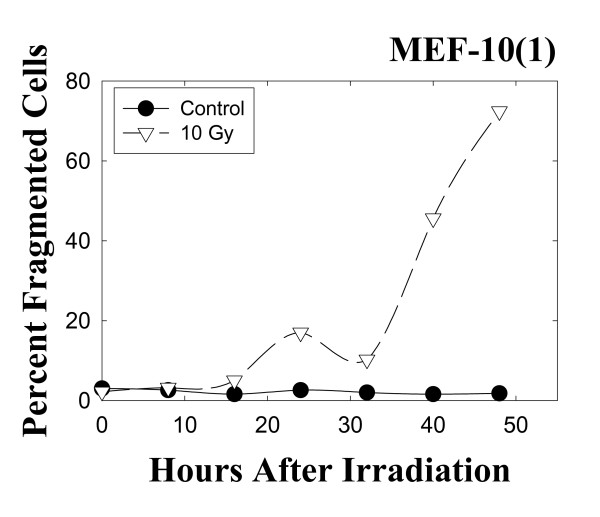
Changes in the frequency of cells with a fragmented nuclear morphology in the mutant p53 cell line MEF 10(1) following 10 Gy γ-irradiation. Intact cells having three or more nuclear fragments were scored as positive for nuclear fragmentation in these experiments, as described in Materials and Methods. Scoring was performed blind.

**Figure 4:**
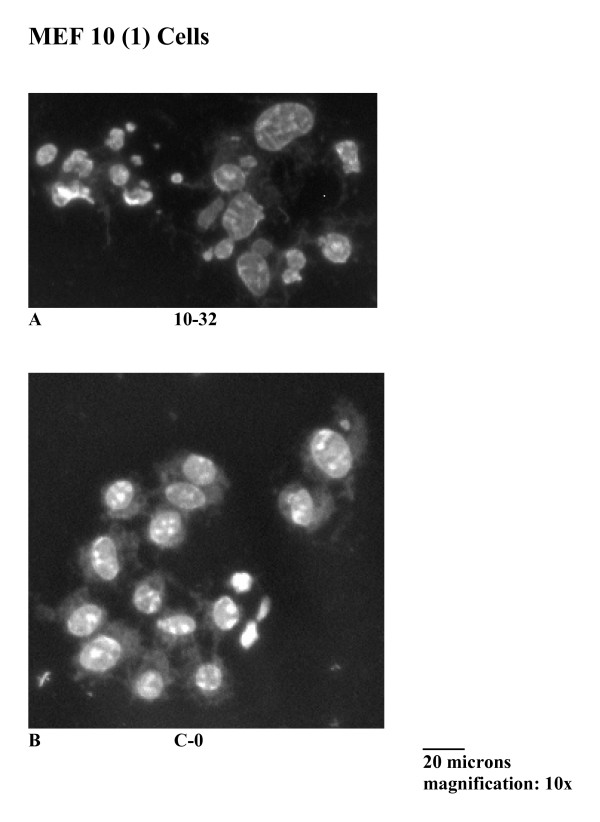
Light microscopy photos of the mutant p53 cell line MEF 10(1) following 10 Gy γ-irradiation. Methanol/glacial acetic acid fixed cells were stained using Hoechst 33258 and visualized using fluorescence microscopy, as described in Materials and Methods. Panel A: morphology of MEF 10(1) cells at 32 hours post-irradiation. Panel B: morphology of sham irradiated MEF 10(1) cells, at time zero.

Figure 3 shows that nuclear fragmentation in this experiment began to rise at about 24 hours post-irradiation. Only intact cells containing three or more fragmented nuclei were scored for these experiments to avoid data interference from possibly present binucleated cells in the irradiated cell population [11]. Nuclear fragmentation is persistent during the time course of this experiment and reaches values of about 80% at 48 hours post-irradiation. Morphological changes in irradiated p53 mutant MEF 10(1) cells are shown in Figure 4. The presence of fragmented nuclei and changes in cell shape are evident for the irradiated population and are hallmarks of MC (Figure 4, panel A; panel B is sham-irradiated control).

**Figure 5:**
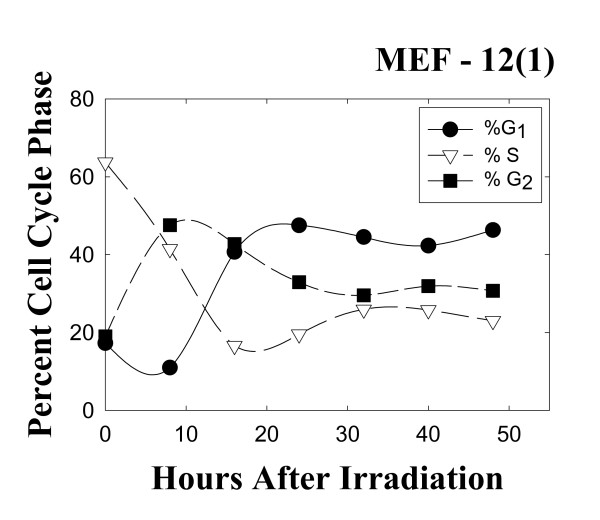
Changes in the cell cycle phase distribution of the wild-type p53 cell line MEF 12(1) following 10 Gy γ-irradiation. Cells were pulse-labeled for 30 min with 10 μM BrdUrd in complete medium at 37°C at the indicated times post-irradiation. Bivariate BrdUrd-PI flow cytometric analysis was performed as described in Materials and Methods. 20000 cells were analyzed at each time point. Closed circles, percentage of cells in G1 phase of the cell cycle; open triangles, percentage of cells in S phase of the cell cycle; closed squares, percentage of cells in G2 phase of the cell cycle.

**Figure 6:**
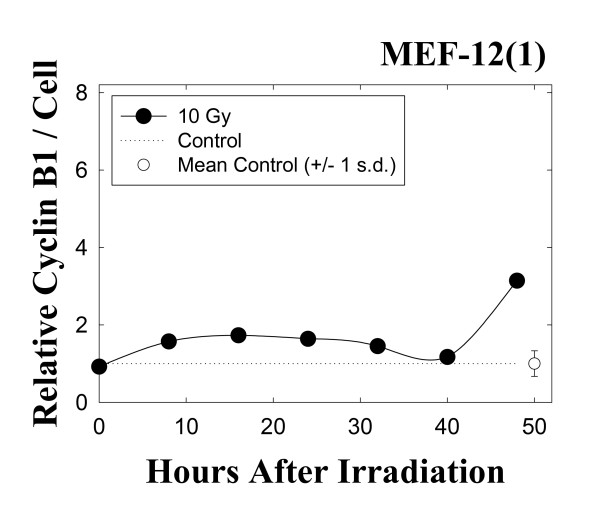
Changes in cellular levels of cyclin B1 in the wild-type p53 cell line MEF 12(1) following 10 Gy γ-irradiation. Cyclin B1 levels were determined using a bivariate flow cytometric assay, as described in Materials and Methods, and are expressed relative to sham-irradiated controls. The open circle at the end of the dotted line shows the mean and standard deviation of cyclin levels in the matched controls over the course of this experiment. Cyclin B1 was detected only in cells in late S and G2 phases.

**Figure 7:**
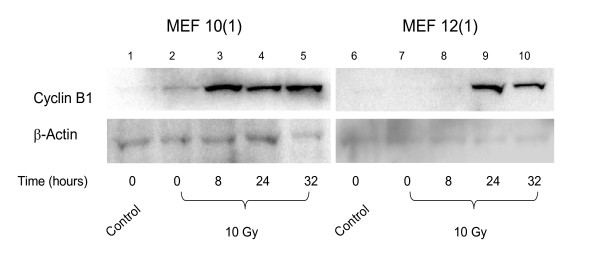
Expression of cyclin B1 in the mutant p53 cell line MEF 10(1) and in the wild-type p53 cell line MEF 12(1) following 10 Gy γ-irradiation. Thirty μg total proteins from whole cell extracts were resolved using SDS-PAGE, and processed as described in Materials and Methods. Immunoblots were visualized using chemiluminesence detection. Lanes 1–5: p53 mutant MEF 10(1) cells: sham-irradiated control at zero time (line 1), followed by 0, 8, 24, and 32 hours post-irradiation samples. Lanes 6–10: wild-type p53 MEF 12(1) cells: sham-irradiated control at zero time (line 6), followed by 0, 8, 24, and 32 hours post-irradiation samples.

**Figure 8:**
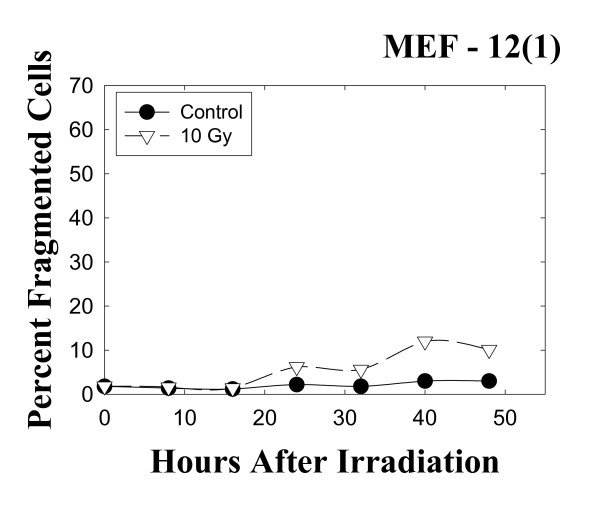
Changes in the frequency of cells with a fragmented nuclear morphology in the wild-type p53 cell line MEF 12(1) following 10 Gy γ-irradiation. Intact cells having three or more nuclear fragments were scored as positive for nuclear fragmentation in these experiments, as described in Materials and Methods. Scoring was performed blind.

**Figure 9:**
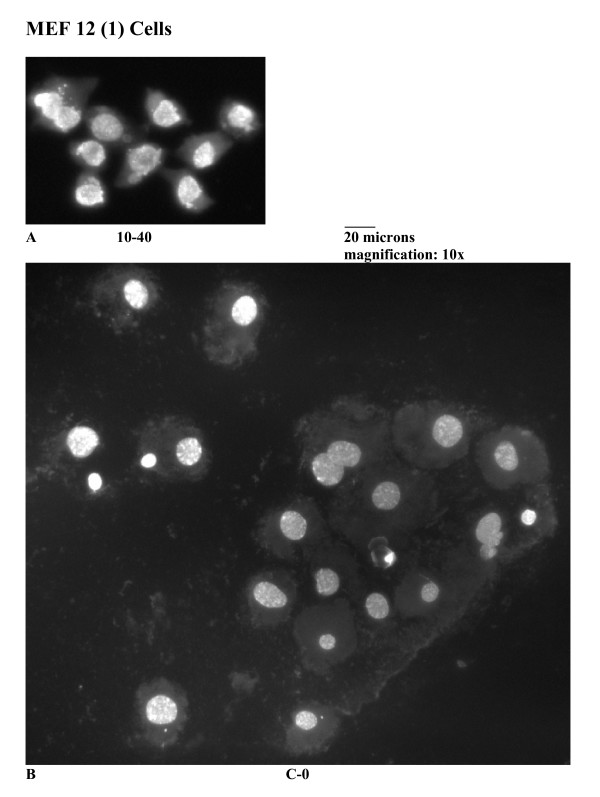
Light microscopy photos of wild-type p53 cell line MEF 12(1) following 10 Gy γ-irradiation. Methanol/glacial acetic acid fixed cells were stained using Hoechst 33258 and visualized using fluorescence microscopy, as described in Material and Methods. Panel A: morphology of MEF 12(1) cells at 40 hours post-irradiation. Panel B: morphology of sham-irradiated MEF 12(1) cells, at time zero.

A different pattern of results is obtained with the wild-type p53 MEF 12(1) cells. The bivariate BrdUrd-PI flow-cytometric analysis of MEF 12(1) cells (Figure 5) shows that following 10 Gy irradiation, similar transient delays late in the cell cycle are present as it is in the mutant p53 MEF 10(1) cells, with the exception that the increase in G2 fraction is observed earlier in this cell line, and this fraction remains elevated for the duration of the experiment, suggesting a persistent G2 arrest. Analysis of cyclin B1 levels, however, showed only a minimal increase in this positive mitotic regulator (Figure 6) in the wild-type p53 cell line. These results are confirmed by western blotting (Figure 7) which shows that cyclin B1 protein levels are increased starting at zero time post-irradiation and remaini elevated there after in the mutant p53 MEF 10(1) cell line, while no protein increase is seen for the first 24 h post-irradiation in the wild-type p53 MEF 12(1) cells. Similarly, minimal nuclear fragmentation was observed post-irradiation in the MEF 12(1) cells (Figure 8). Light microscopy photos of irradiated MEF 12(1) cells also confirm that the morphological features of the irradiated cell population does not greatly differ from the control population (Figure 9, panels A and B), except that a greater number of large cells are observed, probably reflecting the persistent G2 arrest noted in Figure 5. These results suggest an important role for p53 in the induction of MC following irradiation.

## Discussion

Our previous studies of heat- and radiation-induced MC in HeLa cells [20,11,13] have demonstrated that a common feature of the effect of the two modalities is the high accumulation of cyclin B1 that occurs prior to the appearance of MC. Other studies also indicated that overaccumulation of cyclin B1 is a common phenomenon also in other cell lines exposed to radiation [12] as long as p53 is non-functional. Further, we have also shown that in some cell lines, this elevation of cyclin B1 levels persists for several cell generations in the treated population [11]. Although studies have shown that p53 deficiency can lead to the disruption of many cell cycle regulatory pathways [22,29], other reports have demonstrated that effects of p53 on cyclin B1 gene expression alone can contribute significantly to the regulation of the G2/M checkpoint [27,30]. p53 acts to regulate cellular levels of cyclin B1, through a transcriptional repression mechanism which is not completely understood [31,32]. Nevertheless, studies have shown that inhibition of p53-dependent regulation of cyclin B1 transcription results in MC in G2-arrested cells [5,27], and over expression of cyclin B1 can overcome the G2 block [28]. Other studies [33] reported that MC is independent of p21 (Waf-1) and 14-3-3-σ, but p21 can lead to G1 arrest in tetraploid cells produced during MC; and [34] that cells not expressing p21 have an increased number of cells undergoing MC. Thus, it appears that, in addition to those p53-dependent pathways related to cyclin B1/cdc2 activation, at least one pathway has evolved which functions to simply repress cyclin B1 synthesis, and abrogation of this pathway can lead to MC and other subsequent effects on cell cycle regulation.

Control of cyclin B1 levels during a G2 arrest is an important cellular function, because the level of other cell cycle regulators can be effectively reduced simply by mass action in the presence of elevated cyclin B1 protein if their levels are not increased accordingly [28]. We consider it possible that p53 functions to repress radiation-induced MC through its activity as a modulator of the G2 checkpoint mechanisms. On the other hand, it has also been proposed that lack of p53 promotes MC as a mechanism for removing damaged cells from populations following genotoxic stress [35]. The transcriptional repression mechanisms by which p53 mediates a decrease in intracellular levels of cyclin B1 are lost in absence of p53 function; thus, under conditions of genotoxic stress cyclin B1 protein can accumulate, cyclin B1/cdc2 complexes can be activated, the G2 checkpoint can consequently be abrogated and, the cells can enter mitosis prematurely undergoing MC. The generality of this phenomenon as a toxicological mechanism underlying radiation-induced MC has not yet been established in a variety of human tumor cell lines. However, we have demonstrated that MC does occur in human tumor cells such as HeLa cells [11,13,18,19] and PC-3 (prostate cancer) cells (manuscript in preparation), which are both impaired on their p53 functionality. On the contrary, when p53 is functional, such as in the human glioblastoma U87MG cells, MC is not observed (manuscript submitted), consistent with this hypothesis. Thus, p53-mediated effects on cyclin B1 levels may have a dual aspect: tumor cells with functional p53 might be more prone to survive while those with non functional p53 might be driven to undergo MC and die. Defining the mechanisms underlying the role of p53 in MC might lead to strategies to improve clinical radiation response for those human tumors with defects in p53 or p53-related pathways and that avoid radiation-induced apoptosis. If radiation-induced MC is the predominant mode of cell death in p53-deficient cells, clinical interventions designed to enhance its production might not affect surrounding normal tissue, and thus lead to a therapeutic gain.

## Competing interests

The author(s) declare that they have no competing interests.

## Authors' contributions

FI and MAM conceived of the study, participated in its design and coordination, they also drafted the manuscript. FI participated in the FCM measurements and analysis. MAM scored the nuclear fragmentation and took light microscopy photographs. AB and SLP carried out the western blotting experiments; AB also participated in the FCM measurements. EAK carried out the cytology experiments and participated in the FCM measurements. All authors have read and approved the final manuscript.

## References

[B1] Heald R, McLoughlin M, McKeon F (1993). Human wee1 maintains mitotic timing by protecting the nucleus from cytoplasmically activated cdc2 kinase. Cell.

[B2] Gould KL, Nurse P (1989). Tyrosine phosphorylation of the fission cdc2+ protein kinase regulates entry into mitosis. Nature.

[B3] Norbury C, Blow J, Nurse P (1991). Regulatory phosphorylation of the p34cdc2 protein kinase in vertebrates. Embo Journal.

[B4] Flatt PM, Tang LJ, Scatena CD, Szak ST, Pietenpol JA (2000). p53 Regulation of G2 checkpoint is retinoblastoma protein dependent. Mol Cell Biol.

[B5] Hirose Y, Berger MS, Pieper RO (2001). Abrogation of the Chk1-mediated G(2) checkpoint pathway potentiates temozolomide-induced toxicity in a p53-independent manner in human glioblastoma cells. Cancer Res.

[B6] Jackson JR, Gilmartin A, Imburgia C, Winkler JD, Marshall LA, Roshak A (2000). An indolocarbazole inhibitor of human checkpoint kinase (Chk1) abrogates cell cycle arrest caused by DNA damage. Cancer Res.

[B7] Lock RB, Stribinskiene L (1996). Dual modes of death induced by etoposide in human epithelial tumor cells allow Bcl-2 to inhibit apoptosis without affecting clonogenic survival. Cancer Res.

[B8] Yoshikawa R, Kusunoki M, Yanagi H, Noda M, Furuyama JI, Yamamura T, Hashimoto-Tamaoki T (2001). Dual antitumor effects of 5-fluorouracil on the cell cycle in colorectal carcinoma cells: a novel target mechanism concept for pharmacokinetic modulating chemotherapy. Cancer Res.

[B9] Mackey MA, Anolik SL, Roti Roti JL (1992). Cellular mechanisms associated with the lack of chronic thermotolerance expression in HeLa S3 cells. Cancer Res.

[B10] Mackey MA, Morgan WF, Dewey WC (1988). Nuclear fragmentation and premature chromosome condensation induced by heat shock in S-phase Chinese hamster ovary cells. Cancer Res.

[B11] Ianzini F, Mackey MA (1997). Spontaneous premature chromosome condensation and mitotic catastrophe following irradiation of HeLa S3 cells. Intl J Radiat Biol.

[B12] Ianzini F, Cherubini R, Mackey MA (1999). Mitotic catastrophe induced by exposure of V79 Chinese hamster cells to low energy protons. Int J Radiat Biol.

[B13] Mackey MA, Ianzini F (2000). Enhancement of radiation-induced mitotic catastrophe by moderate hyperthermia. Int J Radiat Biol.

[B14] Prieur-Carrillo G, Chu K, Lindqvist J, Dewey WC (2003). Computerized video time-lapse (CVTL) analysis of the fate of giant cells produced by X-irradiating EJ30 human bladder carcinoma cells. Radiat Res.

[B15] Erenpreisa Je, Cragg M, Fringes B, Sharakhov I, Illidge TM (2000). Release of mitotic descendants by giant cells from irradiated Burkitt's lymphoma cell lines. Cell Biol Int.

[B16] Erenpreisa Je, Kalejs M, Ivanov A, Illidge TM, Ianzini F, Kosmacek EA, Mackey MA, Dalmane A, Cragg MS (2005). Genomes segregation in polyploid tumor cells following mitotic catastrophe. Cell Biol Int.

[B17] Ianzini F, Mackey MA (2002). Development of the Large-Scale Digital Cell Analysis System. Radiat Prot Dosimetry.

[B18] Swanson PE, Carroll SB, Zhang XF, Mackey MA (1995). Spontaneous premature chromosome condensation micronucleus formation, and non-apoptotic cell death in heated HeLa S3 cells. Am J Pathol.

[B19] Swanson PE, Zhang XF, Mackey MA (1995). Non-apoptotic cell death in heated HeLa S3 cells – Ultrastructural observations. Lab Invest.

[B20] Mackey MA, Zhang XF, Hunt C, Sullivan S, Blum J, Laszlo A, Roti Roti JL (1996). Uncoupling of M-phase kinase activation from the completion of S phase by heat shock. Cancer Res.

[B21] Canman CE (2001). Replication checkpoint: preventing mitotic catastrophe. Curr Biol.

[B22] Chan TA, Hermeking H, Lengauer C, Kinzler KW, Vogelstein B (1999). 14-3-3σ is required to prevent mitotic catastrophe after DAN damage. Nature.

[B23] Sato N, Mizumoto K, Nakamura M, Tanaka M (2000). Radiation-induced centrosome overduplication and multiple mitotic spindles in human tumor cells. Exp Cell Res.

[B24] Holmfeldt P, Larsson N, Segerman B, Howell B, Morabito J, Cassimeris L, Gullberg M (2001). The catastrophe-promoting activity of ectopic Op18/stathmin is required for disruption of mitotic spindles but not interphase microtubules. Mol Biol Cell.

[B25] Pines J, Hunter T (1989). Isolation of a human cyclin cDNA: Evidence for cyclin mRNA and protein regulation in the cell cycle and for interaction with p34cdc2. Cell.

[B26] Pines J, Hunter T (1991). Human, cyclins A and B1 are differentially located in the cell and undergo cell cycle-dependent nuclear transport. J Cell Biol.

[B27] Innocente SA, Abrahamson JLA, Cogswell JP, Lee JM (1999). p53 regulates a G2 checkpoint through cyclin B1. Proc Natl Acad Sci USA.

[B28] Murray AW (1992). Creative blocks: cell cycle checkpoints and feedback controls. Nature.

[B29] Passalaris TM, Benanti JA, Gewin L, Kiyono T, Galloway DA (1999). The G2 checkpoint is maintained by redundant pathways. Mol Cell Biol.

[B30] Park M, Chae HD, Yun J, Jung M, Kim Y-S, Kim S-H, Moon HH, Shin DY (2000). Constitutive activation of cyclin B1-associated cdc2 kinase overrides p53-mediated G2/M arrest. Cancer Res.

[B31] Taylor WR, Agarwal ML, Agarwal A, Stacey DW, Stark GR (1999). p53 inhibits entry into mitosis when DNA synthesis is blocked. Oncogene.

[B32] Taylor WR, Stark GR (2001). Regulation of the G2/M transition by p53. Oncogene.

[B33] Andreassen PR, Lacroix FB, Lohez OD, Margolis RL (2001). Neither p21(WAF1) nor 14-3-3sigma revents G(2) progression to mitotic catastrophe in human colon carcinoma cells after DNA damage, but p21(WAF1) induces stable G(1) arrest in resulting tetraploid cells. Cancer Res.

[B34] Chu K, Teele N, Dewey MW, Albright N, Dewey WC (2004). Computerized video time lapse study of cell cycle delay and arrest, mitotic catastrophe, apoptosis and clonogenic survival in irradiated 14-3-3σ and CDKN1A (p21) knockout cell lines. Radiat Res.

[B35] Vogelstein B, Kinzler KW (2001). Achille's heel of cancer?. Nature.

